# Shannon Entropy of Ramsey Graphs with up to Six Vertices

**DOI:** 10.3390/e25101427

**Published:** 2023-10-09

**Authors:** Mark Frenkel, Shraga Shoval, Edward Bormashenko

**Affiliations:** 1 Chemical Engineering Department, Engineering Faculty, Ariel University, P.O.B. 3, Ariel 407000, Israel; markfr@ariel.ac.il; 2 Department of Industrial Engineering and Management, Faculty of Engineering, Ariel University, P.O.B. 3, Ariel 407000, Israel; shraga@ariel.ac.il

**Keywords:** complete graph, Shannon entropy, bi-colored graph, Ramsey theorem, Ramsey number, Voronoi tessellation

## Abstract

Shannon entropy quantifying bi-colored Ramsey complete graphs is introduced and calculated for complete graphs containing up to six vertices. Complete graphs in which vertices are connected with two types of links, labeled as α-links and *β*-links, are considered. Shannon entropy is introduced according to the classical Shannon formula considering the fractions of monochromatic convex α-colored polygons with *n α*-sides or edges, and the fraction of monochromatic β-colored convex polygons with *m β*-sides in the given complete graph. The introduced Shannon entropy is insensitive to the exact shape of the polygons, but it is sensitive to the distribution of monochromatic polygons in a given complete graph. The introduced Shannon entropies $S_{\alpha }$ and $S_{\beta }$ are interpreted as follows: $S_{\alpha }$ is interpreted as an average uncertainty to find the green α−polygon in the given graph; $S_{\beta }$ is, in turn, an average uncertainty to find the red β−polygon in the same graph. The re-shaping of the Ramsey theorem in terms of the Shannon entropy is suggested. Generalization for multi-colored complete graphs is proposed. Various measures quantifying the Shannon entropy of the entire complete bi-colored graphs are suggested. Physical interpretations of the suggested Shannon entropies are discussed.

## 1. Introduction

The intriguing history of the birth and development of the Ramsey theory is surveyed in detail in ref. [1]. Frank Plumpton Ramsey was a British logician, mathematician, and thinker who made major contributions to various fields of knowledge before his death at the age of 26. His brother Michael once said about Frank Ramsey, “He was interested in almost everything” [1]. In our paper we address the sub-field of the graph theory, which is known as the “Ramsey theory”, which deals with the specific kind of graphs, namely: complete graphs. A complete graph is a graph in which each pair of graph vertices is connected by an edge/link. The classical problem considered by the Ramsey theory is the so-called “party problem”, which predicts the minimum number of guests $R\left(m,n\right)$ that must be invited so that at least *m* of the guests will be acquainted with each other, or at least *n* of them will not be familiar with each other [2,3,4,5,6,7]. In this case $R\left(m,n\right)$ is known as a Ramsey number [7,8,9,10,11]. A classical result in Ramsey theory states that if some mathematical structure/graph is separated into finitely many sub-parts, then one of the sub-parts necessarily must contain a substructure/graph of the given type.

Let us discuss first the infinite Ramsey theorem in a rigorous way: let us think about all the positive integers, and imagine joining every pair with a line. Every pair of positive integers is joined by a line. Let us denote the emerging graph as $K_{\infty }$. Now, we color each line either red or green. The infinite two-color Ramsey theorem states that no matter how we two-color the edges in $K_{\infty },$ it will always be possible to find infinitely many points that are all connected by the same color. In other words: consider $K_{\infty }$ which is the complete graph whose vertex set is countably infinite; every two-colored $K_{\infty }$ must contain a countably infinite monochromatic complete graph. More general formulation of the infinite Ramsey theorem for multi-colored graphs, states that if we color each edge of an infinite, complete graph, with one out of finitely many prescribed colors, then there is an infinite, complete monochromatic subgraph. That is, an infinite set of vertices such that all edges among them have the same color. And the more general re-shaping of the infinite Ramsey theorem states that, if we split an infinite object with a certain regularity property (such a set containing arbitrary long arithmetical progressions) into two parts, one infinite part will exhibit this property too [7].

The finite Ramsey theorem states that there exists *R(m)* which is the smallest *n* such that, for all bi-colorings of $K_{n}$, there is a homogeneous set of size *m*. In its graph-theory form, Ramsey’s theorem states that there exist monochromatic cliques in any edge labelling (with colors) of a sufficiently large complete graph [7,8,9,10,11]. A clique in graph theory is a subset of the vertices of a graph (undirected) such that every two distinct vertices in the clique are adjacent; that is, its induced subgraph is complete.

In the graph theory, a complete graph is a simple undirected graph in which every pair of different vertices is connected by a unique edge [7,8,9,10,11]. Complete graphs emerge in various fields of economic science, physics, and engineering. For example, cyclic molecules may be seen as a system of particles interconnected with springs of various kinds [12]. Sets of thermodynamic states form complete graphs [13]. Interacting particles/dipoles may be seen as complete graphs [14]. Ramsey theory was successfully applied for the theory of communication and decision making [15]. Thus, numerous applications of Ramsey theory are foreseen.

## 2. Shannon Entropy and Voronoi Tessellations

The present paper is devoted to the quantitative characterization of bi-colored Ramsey graphs. We propose to carry out this quantification with Shannon entropy, successfully applied for the characterization of Voronoi tessellations [16,17,18,19]. Voronoi tessellation is a partitioning of an infinite plane into regions based on the distance to a specified discrete set of points (called seeds or nuclei) [16,17,18,19,20,21]. Voronoi tessellation divides a plane into polygons, known as cells, which surround each point/seed, consisting of the region of the plane nearer to that point than any other. It is noteworthy that the Voronoi tessellation actually was introduced first by Descartes [19]. Voronoi tessellations are usually quantified with the so-called Shannon entropy, which was discovered in 1948 by Claude Shannon, which is used as a measure of information, of uncertainty, and unlikelihood [22,23,24]. This measure is defined for any given probability distribution [22,23,24]. The quantity introduced by Shannon has the same mathematical form as the entropy in statistical mechanics, thus, he labeled this measure, as allegedly suggested by von Neumann: “entropy”. For any random variable *X*, characterized by a probability distribution $p_{1},p_{2}\ldots p_{n}$, the Shannon entropy denoted *H* is defined as follows:

(1) $$H=-\sum_{i=1}^{n}{p_{i}log}_{2}p_{i}$$

If *X* is an experiment having *n* outcomes, then $p_{i}$ is the probability associated with the occurrence of the outcome *i*. For the extended discussion of the accurate interpretation of the Shannon entropy see ref. [23]. Equation (1) resembles the definition of the Boltzmann/thermodynamic entropy $S_{B}$, which is a logarithmic measure of the number of system states with significant probability of being occupied, given by $S_{B}=-k_{B}\sum_{n}p_{i}lnp_{i}$, where $k_{B}$ is the Boltzmann constant $p_{i}$ p i is the probability that the system is in *i i*-th state, usually given by the Boltzmann distribution [23]. Regrettably, numerous misinterpretations of the Shannon entropy, arising from the resemblance of the aforementioned formulae, appeared in the scientific literature [23].

Shannon Entropy, as applied to Voronoi tessellations, may be seen as a measure of “ordering” in a given tessellation [17,18,19,20,21,22]. Controversies of such an understanding of the Shannon entropy are discussed in ref. [22]. For any given set of points corresponding to the Voronoi tessellation diagram, the Shannon entropy (also known as the Shannon measure of information), denoted *S*, is defined by Equation (2):

(2) $$S=-\sum_{n}P_{n}lnP_{n},$$

where $P_{n}$ is the fraction of polygons with *n* sides or edges in a given Voronoi tessellation [16,17,18,19,20,21,22]. We demonstrate how the Shannon entropy may be introduced for Ramsey bi-colored complete graphs [23,24].

## 3. Shannon Entropy of Ramsey Graphs

### 3.1. Shannon Entropy of Complete Bi-Color Graphs Built of Three Vertices

**Figure 1:**
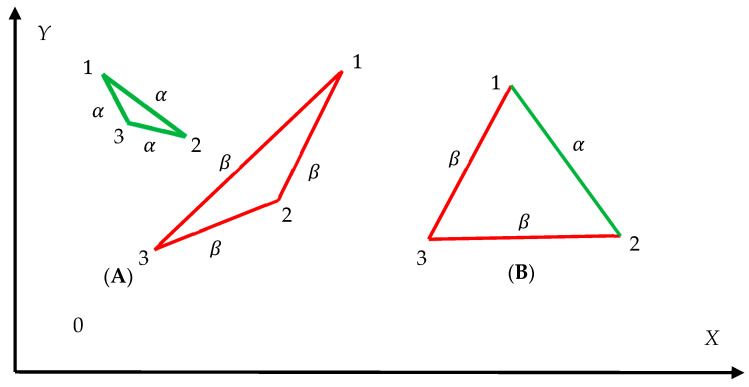
Simplest mono-colored and bi-colored graphs built of three vertices and three edges are depicted. (**A**) monochromatic triangles built of green α-links and red β-links are shown. (**B**) Bi-color triangle graph is depicted.

We start from the simplest complete graphs forming triangles depicted in Figure 1. Vertices of the graphs are denoted “123”. Vertices are connected with two kinds of links (edges) labeled α-links and β-links: α-links are colored with green and β-links are colored with red (see Figure 1). An edge/link in graph theory is (together with vertices) one of the two basic units out of which graphs are constructed. Each edge has two vertices to which it is attached, called its endpoints. We connect the vertices of the triangle with red or green links, as shown in Figure 1. Thus, complete, bi-colored, or mono-colored graphs, addressed by Ramsey theory, emerge. Two situations are possible, the graphs are built of edges of the same kind as depicted in inset A of Figure 1, and graphs built of the edges of different kinds as shown in inset B of Figure 1.

Let us introduce the Shannon entropies of the graphs according to Equations (3) and (4) (compare with the classical definition of the Shannon entropy defined with Equation (2)):

(3) $$S_{\alpha }=-\sum_{n}P_{n\alpha }lnP_{na},\;n\geq 3$$

(4) $$S_{\beta }=-\sum_{i}P_{i\beta }lnP_{i\beta },\;i\geq 3$$

where $P_{n\alpha }$ is the fraction of monochromatic α-colored convex polygons with *n α*-sides or edges (green edges), and $P_{i\beta }$ is the fraction of monochromatic convex β-colored polygons with *I* β-sides or edges (red edges) in a given complete graph. Sampling of polygons is carried out separately from the green and red subsets of convex polygons. Thus, a pair of Shannon entropies $\left(S_{\alpha },S_{\beta }\right)$ corresponds to any complete bi- or mono-colored complete graph. The introduced Shannon entropies $\left(S_{\alpha },S_{\beta }\right)$ are completely prescribed by the given distribution of polygons in the addressed graph. Let us illustrate this idea with the simplest triangle complete graphs, presented in Figure 1. We start from the green monochromatic triangle shown in inset A of Figure 1. For this graph, we derive: $P_{3\alpha }=1,\;\;for\;n=3,\;\;P_{n\alpha }=0\;for\;n\neq 3$; $P_{i\beta }=0\;\mathrm{for}\mathrm{any}\;i\geq 3.$ Thus, according to Equation (3), the both of the Shannon entropies $S_{\alpha }$ and $S_{\beta }$ equal zero. Now we introduce the following notation:$\;S_{\alpha }=0$, $S_{\beta }=\tilde{0}$. This notation enables distinguishing between zero Shannon entropies emerging from the situations when $P_{n}=0$ and $P_{n}=1$, appearing in Equations (3) and (4), take place; namely $S=0,\;when\;P_{n}=1$ for a given n≥3, and $S=\tilde{0},\;when\;P_{n}=0$ for any n≥3. This distinguishing is useful for separating between various mathematical/physical cases, illustrated with Figure 1, arising from the essentially different coloring of the graph [23]. Thus, for the green monochromatic triangles shown in insert A of Figure 1, we derive:

(5) $$\left(S_{\alpha },S_{\beta }\right)=\left(0,\tilde{0}\right)$$

In turn, for the red monochromatic triangle, depicted in Figure 1, we obtain:

(6) $$\left(S_{\alpha },S_{\beta }\right)=\left(\tilde{0},0\right)$$

Graph depicted in inset B of Figure 1 demonstrate the bi-chromatic triangle. In this graph $P_{n\alpha }=0\;\mathrm{for}\mathrm{any}\;n\geq 3$, $P_{i\beta }=0\;\mathrm{for}\mathrm{any}\;i\geq 3$. Thus, according to the introduced notation, the Shannon entropy corresponding to this graph is given by Equation (7).

(7) $$\left(S_{\alpha },S_{\beta }\right)=\left(\tilde{0},\tilde{0}\right)$$

Thus, the suggested notation enables the exact quantification of various graphs, shown in Figure 1. Moreover, the introduced notation will enable the elegant re-shaping of the Ramsey theory in the terms that will be demonstrated below.

### 3.2. Shannon Entropy of Complete Bi-Color Graphs Built of Four and Five Vertices

**Figure 2:**
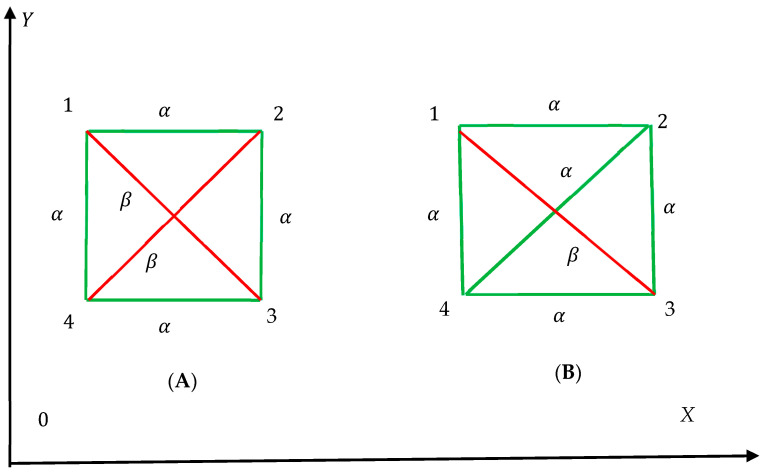
Complete bi-colored graphs, built of four vertices and six edges are depicted. α links are depicted in green; β links are shown in red. (**A**). The single green quadrangle is recognized. $S_{tot}=0$, as calculated with Equation (7). (**B**). One monochromatic green quadrangle “1234” and two monochromatic green triangles “124” and ”234” are recognized. $S_{tot}=0.64$.

Now consider the complete bi-color graph, built of four vertices and six edges, depicted in inset A of Figure 2. Again, α-links (green) and β-links (red) are present in the graph. We recognize the single monochromatic green quadrangle in the graph. Thus, $P_{4\alpha }=1,\;P_{n\alpha }=0,\;n\neq 4$. Thus, we calculate with Equation (3): $S_{\alpha }=0$. There is no monochromatic red polygon in inset A of Figure 2, hence $P_{i\beta }=0\;\mathrm{for}\mathrm{any}\;i\geq 3$ and according to Equation (4), $S_{\beta }=\tilde{0}$. Finally, the pair of Shannon entropies of the complete bicolor graph, shown in inset A of Figure 2 is given by: $\left(S_{\alpha },S_{\beta }\right)=\left(0,\tilde{0}\right)$. Now we address the complete bi-color graph, depicted in inset B of Figure 2. We recognize one monochromatic, green quadrangle “1234” and two monochromatic green triangles “124” and ”234”. No monochromatic red polygons are recognized. The Shannon entropies are exhaustively defined by the aforementioned distribution of monochromatic convex polygons in the given graph. Thus, we calculate: $P_{4\alpha }=\frac{1}{3},\;P_{3a}=\frac{2}{3}$. According to Equation (3) we obtain: $S_{\alpha }=-\left(\frac{1}{3}ln\frac{1}{3}+\frac{2}{3}ln\frac{2}{3}\right)=0.64$. There is no monochromatic red polygon in inset A of Figure 2, hence $P_{i\beta }=0\;\mathrm{for}\mathrm{any}\;i\geq 3$ and according to Equation (4), $S_{\beta }=\tilde{0}$ takes place for this graph. Finally, the Shannon entropies for the graph shown in inset B of Figure 2 are given by: $\left(S_{\alpha },S_{\beta }\right)=\left(0.64,\tilde{0}\right).$

What is the meaning of the calculated values? We keep the interpretation of Shannon entropy, suggested and discussed in detail in ref. [23]. According to this interpretation $S_{\alpha }$ is an average uncertainty to find the green α−polygon in the given graph, $S_{\beta }$ is, in turn, an average uncertainty to find the red β−polygon in the same graph.

The total Shannon Entropy of the given bi-colored complete graph may be introduced with Equation (8), exploiting Equations (3) and (4).

(8) $${S_{tot}=S}_{\alpha }+S_{\beta }=-\sum_{n}P_{n\alpha }lnP_{na}-\sum_{m}P_{m\beta }lnP_{m\beta }$$

where $P_{n\alpha }$ is the fraction of monochromatic α-colored convex polygons with *n α*-sides or edges (green edges), and $P_{i\beta }$ is the fraction of monochromatic convex β-colored polygons with *i* β-sides or edges (red edges) in a given complete graph. The total Shannon entropy is defined by the prescribed distribution of monochromatic convex polygons in the given complete graph. Again, sampling of polygons is carried out separately from the green and red subsets of convex polygons. The total Shannon Entropy of the graph depicted in the inset A of Figure 2 is zero; whereas the total entropy of the graph shown in inset B of Figure 2 is $S_{tot}=0.64.$ What is the meaning of these values? An average uncertainty to find the mono-colored polygon (of any color) in inset A is zero; whereas an average uncertainty to find the mono-colored polygon (of any color) in inset B equals 0.64. In other words, $S_{tot}$ quantifies the average unlikelihood, or unexpectedness to find the mono-colored polygon in the given graph [23]. $S_{tot}$ also may be referred to as a measure of the amount of information contained in the given bi-colored graph, associated with a given probability distribution of finding mono-colored polygons within a given graph, when the sampling of polygons is performed separately from the green and red subsets of polygons [23]. It was demonstrated that the Shannon entropy $S_{tot}$ is a measure of information associated with the entire distributions of monochromatic polygons, not with the individual probabilities [23].

**Figure 3:**
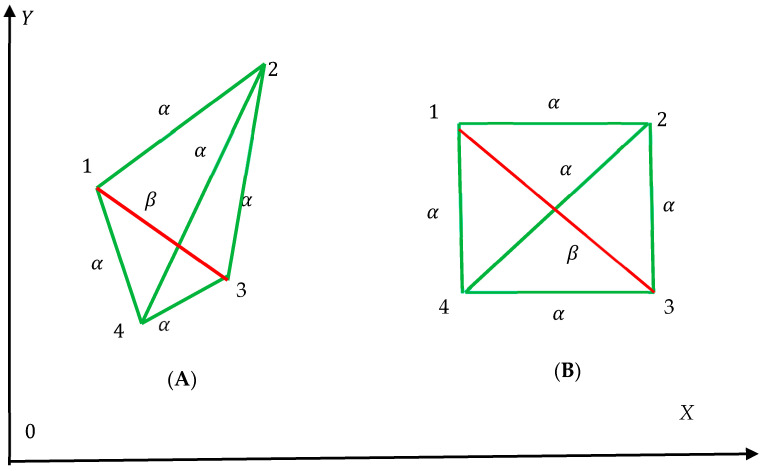
Insensitivity of the Ramsey bi-colored complete graphs to their shapes is demonstrated. Graphs depicted in insets (**A**) a (**B**) are characterized by the same values of the Shannon entropies introduced with Equations (3), (4) and (8). Only the polygon-type distribution impacts the values of the Shannon entropies. $S_{tot}=0.64$ for both graphs.

Two peculiarities of the Shannon entropy defined for the Ramsey graphs with Equations (3), (4) and (8) are noteworthy:

(i) The Shannon entropies are insensitive to the exact shapes of the polygons appearing in the graph (only the number of polygon sides is taken into account), as illustrated in Figure 3. The graphs depicted in insets Figure 2A,B are quantified with the same values of the Shannon entropies introduced with Equations (3) and (4) and Equation (8). Only the polygon-type distribution influences the values of the Shannon entropies.
(ii) Consider now the patterns built of *N* bicolored graphs, presented in Figure 3A,B. The entire Shannon entropy of the patterns will be equal to that of the single graph, namely $S_{tot}=0.64,$ and is independent on the number of the elementary cells/Ramsey graphs. Thus, the Shannon entropy is the intensive property of the pattern in contrast to the well-known Boltzmann entropy. Now address the pattern built of *N* bicolored graphs, presented in Figure 2A. The entire Shannon entropy of the pattern, defined with Equation (8) is zero, $S_{tot}=0$ and it is independent of the number *N*.

It will be also instructive to calculate the Shannon entropy of the mono-colored complete graph built of four vertices, such as those depicted in Figure 2 and Figure 3. Consider the complete graph built of four vertices forming a convex quadrangle, connected with green links only. The complete graph in this case comprises four triangles and one quadrangle. Thus, the Shannon energy is calculated with Equation (3) as $S_{\alpha }=-\left(\frac{1}{5}ln\frac{1}{5}+\frac{4}{5}ln\frac{4}{5}\right)=0.496.$ Finally, the Shannon entropies mono-colored complete graph, forming a convex quadrangle is given by: $\left(S_{\alpha },S_{\beta }\right)=\left(0.496,\tilde{0}\right),\;\;S_{tot}=0.496.$ The minimal possible Shannon entropy quantifying graphs built of four vertices corresponds to the graph, depicted in inset A of Figure 2$,\;\left(S_{\alpha },S_{\beta }\right)=\left(0,\tilde{0}\right),\;S_{tot}=0$.

**Figure 4:**
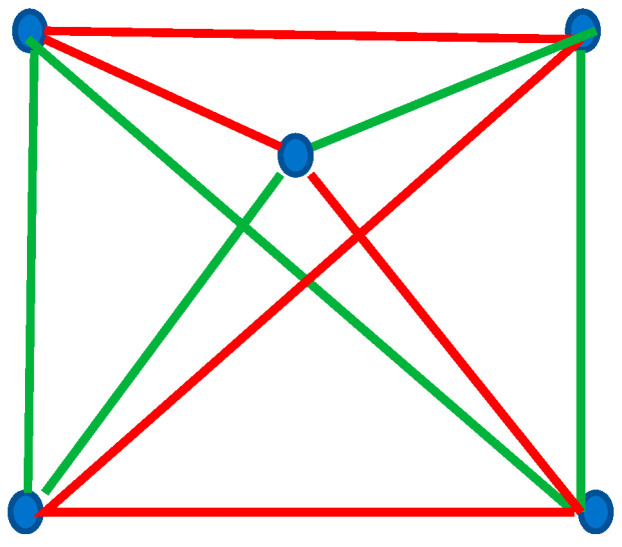
Complete bi-colored graph built of five vertices is shown. No monochromatic triangle or polygons are recognized in the graph. The Shannon entropies of this graph are zero, according to the introduced notation: $\left(S_{\alpha },S_{\beta }\right)=\left(\tilde{0},\tilde{0}\right),\;S_{tot}=0$.

Now consider the Shannon Entropy of the complete bi-colored graph built of five vertices depicted in Figure 4. This graph does not contain any mono-colored triangle or polygon. This is possible according to the Ramsey theorem; indeed, the Ramsey number $R\left(3,3\right)=6$. Thus, the Shannon tntropies of this graph are zero according to the introduced notation: $\left(S_{\alpha },S_{\beta }\right)=\left(\tilde{0},\tilde{0}\right),\;S_{tot}=0$.

### 3.3. Shannon Entropy of Complete Bi-Color Graphs Built of Six Vertices

**Figure 5:**
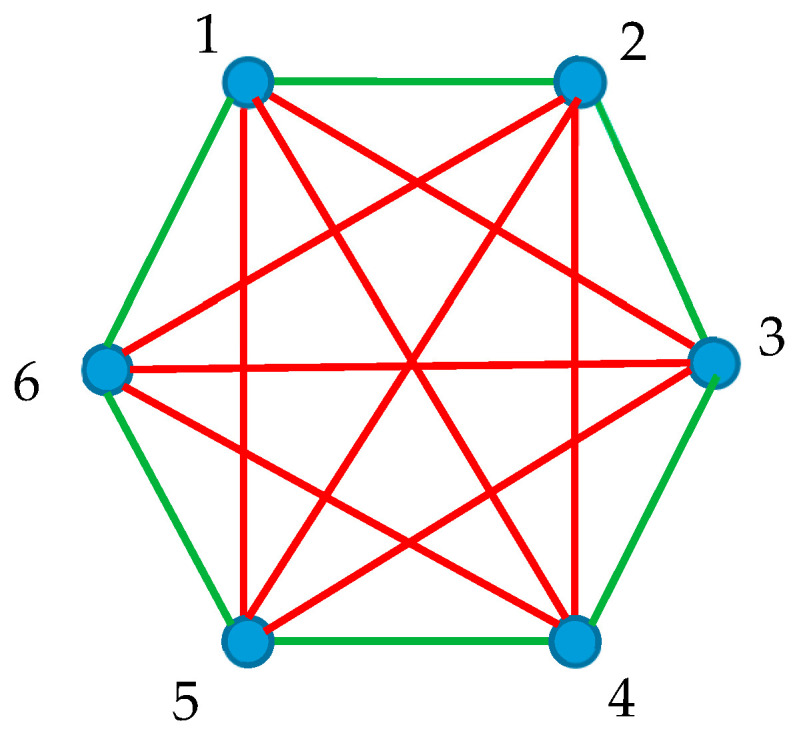
Complete Ramsey bi-colored graph built of six vertices is shown. One green hexagon and two red triangles (“135 and “246”) are recognized in the graph. $\left(S_{\alpha },S_{\beta }\right)=\left(0,0\right)$ is true for the graph.

Consider the complete bi-colored graph built of six vertices depicted in Figure 5. One green α-hexagon and two red β-triangles (namely “135” and “246”) are recognized in the graph. We calculate $P_{4\alpha }=1,\;P_{3\beta }=1$, Thus, the pair of Shannon Entropies, calculated with Equations (3) and (4) of the graph, is given by $\left(S_{\alpha },S_{\beta }\right)=\left(0,0\right)$. In other words, the average uncertainties to find green or red polygons within the graph, shown in Figure 5, are zero.

The introduced Shannon entropy of the complete bi-colored graphs enables the re-shaping of the Ramsey theorem for the graphs built of six vertices. The Ramsey number $R\left(3,3\right)=6$, this means that at least one monochromatic triangle will be inevitably present in any bicolored complete graph built of six vertices. In other words, the complete bi-colored graph, built of six vertices, for which $\left(S_{\alpha },S_{\beta }\right)=\left(\tilde{0},\tilde{0}\right)$ takes place does not exist. The total Shannon entropy of the graph calculated with Equation 8 is zero. Thus, we come to the re-shaping of the Ramsey theorem in the terms of the Shannon entropy.

**Theorem** **1.** *Consider a complete bi-colored graph containing six vertices. The graph is built of two kinds of links, labeled correspondingly the α- and β-links. The Shannon entropies of the graphs are defined according to* $S_{\alpha }=-\sum_{n}P_{n\alpha }lnP_{na},\;n\geq 3$*;* $S_{\beta }=-\sum_{i}P_{i\beta }lnP_{i\beta }$*,* i≥3*, where* $P_{n\alpha }$ *is the fraction of monochromatic convex* α*-colored polygons with nα-sides or edges, and* $P_{i\beta }$ *is the fraction of monochromatic convex * β-colored *polygons with i* β*-sides or edges. Assume that* $S_{\alpha }=0$ *for the graphs for which* $P_{n\alpha }=1$ *is true for a given* n>3, *and* $S_{\alpha }=\tilde{0}$ *for the graphs for which* $P_{n\alpha }=0$ *for any* n>3 *takes place. Correspondingly,* $S_{\beta }=0$*, for the graphs for which,* $P_{i\beta }=1$ *is true for a given* i>3, *and* $S_{\beta }=\tilde{0}$ *for the graphs for which* $P_{i\beta }=0$ *for any* i>3  *is assumed. No bi-coloring of the graph exists for which* $\left(S_{\alpha },S_{\beta }\right)=\left(\tilde{0},\tilde{0}\right)$ *is true.*

Now we demonstrate one more, alternative possibility to introduce the total Shannon Entropy of the bi-colored Ramsey complete graph, labeled $\hat{S}$ and introduced with Equation (9):

(9) $$\hat{S}=-\sum_{k}P_{k}lnP_{k},k\geq 3,$$

where $P_{k}$ is the fraction of monochromatic polygons (whatever red or green ones) in the given complete graph. Now, sampling takes place over the entire set of monochromatic polygons, whatever their colors. Let us calculate $\hat{S}$ for the graph depicted in Figure 5. This graph comprises three monochromatic polygons, namely two red triangles and one green hexagon. Thus, $\hat{S}$ for this graph is calculated as follows: $\hat{S}=-\left(\frac{1}{3}ln\frac{1}{3}+\frac{2}{3}ln\frac{2}{3}\right)=0.64$. What is the meaning of $\hat{S}?$ $\hat{S}$ is adequately interpreted as the average uncertainty to find the monochromatic polygon (whatever its color) within the given complete bi-colored graph. Consider the graphs shown in Figure 1, Figure 2 and Figure 3. $\hat{S}=0$ for all of the graphs, shown in Figure 1. We recognize a single green quadrangle for the graph shown in inset A of Figure 2; thus, in this case, $\hat{S}=0$. For the graph, shown in inset B of Figure 2 we calculate: $\hat{S}=-\left(\frac{1}{3}ln\frac{1}{3}+\frac{2}{3}ln\frac{2}{3}\right)=0.64.$ The value of $\hat{S}$ is exhaustively defined by the distribution of polygons in the given complete graph, and it is independent on the exact shapes of the polygons; $\hat{S}=0.64$ for both of graphs, depicted in Figure 3. $\hat{S}=0$ for the graph presented in Figure 4; indeed, no monochromatic polygon is recognized in the graph.

## 4. Discussion

(i) The paper presents the synthesis of three mathematical ideas, namely: Ramsey complete graphs, Voronoi tessellations, and the Shannon measure of information. We demonstrate that the Shannon entropy may be introduced for the bi-colored graphs in a way similar to that in which it is defined for the Voronoi diagrams/tessellations. For *p*-colored graphs a set of Shannon entropies $\left(S_{1},S_{2}\ldots S_{r}\ldots S_{p}\right),$ should be introduced where $S_{r}$ is given by:
  (10) $$S_{r}=-\sum_{n}P_{nr}lnP_{nr},n\geq 3;1\leq r\leq p$$
  where $P_{nr}$ is the fraction of monochromatic *r*-colored polygons with *n* edges. Thus, the matrix of probabilities $P_{nr}$ emerges. Obviously ${0\leq P}_{nr}\leq 1,\;n\geq 3;\;1\leq r\leq p$ is true.
(ii) Graphs depicted in Figure 1, Figure 2 and Figure 3 are embedded into *XOZ* coordinate frames. In our recent paper we suggested coloring of the graphs dependent on the orientation of the coordinate axes [25]. Thus, the introduced Shannon Entropies will be also dependent of the orientation of the coordinate axes. We plan to study this dependence in our future investigations.

Let us discuss the physical/chemical meaning/interpretation of the introduced Shannon entropies $\left(S_{\alpha },S_{\beta }\right).$ The complete bi-colored graph, presented in Figure 5, may be interpreted as a scheme of a cyclic molecule, in which two kinds of chemical bonds depicted with green (α) links and red $\left(\beta \right)$ links/edges are present [12]. In this case, the Shannon entropies $\left(S_{\alpha },S_{\beta }\right)$ are seen as averaged uncertainties to find *α* or *β* cyclic sub-structures within the molecule. The presence of cyclic structures will influence the vibrational spectrum of the molecule [12].

Another physical interpretation of the graph, shown in Figure 5, emerges, when the vertices of the complete graph are treated as interacting particles [14]. In this case, *α*-links may correspond to the attraction between the particles, and *β*-links may correspond, in turn, to repulsion between interacting entities (for example, electric or magnetic dipoles) [14]. In this situation, the Shannon Entropies $\left(S_{\alpha },S_{\beta }\right)$ are interpreted as an averaged uncertainties to find *α* or *β* sub-structures, in which the entities interact only by attraction or repulsive forces within an entire set of particles. This reasoning may be important for predicting the elementary cell of crystals built of electric or magnetic dipoles [14].

We do not suggest the algorithm enabling the calculation of the introduced Shannon entropies $\left(S_{\alpha },S_{\beta }\right)$ for a given Ramsey graph, and this fact stipulates a weakness of the proposed approach. The algorithmic calculation of Ramsey numbers also remains an unsolved problem, in spite of the progress in this field reported recently [26,27,28,29,30].

## 5. Conclusions

We conclude that the Shannon entropy/measure of information may be successfully introduced for the Ramsey complete bi-colored graphs. We addressed the complete bi-colored graphs containing up to six vertices. Shannon entropy is introduced according to the classical Shannon formula considering the fractions of monochromatic convex α-colored polygons with *n α*-sides or edges, and the fraction of monochromatic β−colored convex polygons with *m β*-sides in the given complete graph. The introduced Shannon entropies $S_{\alpha }$ and $S_{\beta }$ are interpreted as follows: $S_{\alpha }$ is seen as an average uncertainty to find the green, convex α−polygon in a subset of α polygons of the given graph; $S_{\beta }$ is, in turn, an average uncertainty to find the red, convex β−polygon in the subset of *β*-polygons of the same graph. The sampling of polygons is carried out separately from the green (*α*) and red (*β*) subsets of convex polygons. The introduced Shannon entropies resemble the Shannon entropy of Voronoi diagrams, in which the fractions of *n*-sided polygons in a given Voronoi tessellation yield the Shannon entropy of the entire diagram. Introduced Shannon entropy is insensitive to the exact shape of the polygons in the graph, being sensitive to the distribution of monochromatic polygons in a given graph. The re-shaping of the Ramsey theorem in terms of Shannon entropy is presented. The complete bi-colored graph, built of six vertices, for which $\left(S_{\alpha },S_{\beta }\right)=\left(\tilde{0},\tilde{0}\right)$ takes place does not exist. The alternative ways of defining of the total Shannon entropy of bi-colored graphs are suggested. The additive pathway of the calculation of the total Shannon entropy of the bi-colored graph implies $S_{tot}=S_{\alpha }+S_{\beta }$, this definition means that $S_{tot}$ quantifies the average unlikelihood, or unexpectedness to find the mono-colored convex polygon (of any of colors) in the given complete graph, when sampling of polygons is carried out separately from the green and red subsets of convex polygons. The generalization of the suggested approach for multi-colored complete graphs is introduced. We also considered the patterns built of *N* bi-colored graphs; the introduced Shannon entropy turns out to be the intensive property of the pattern, being independent on the number *N.* This differs the introduced Shannon entropy from the Boltzmann entropy, which is an extensive characteristic of the sample.

The complete bi-colored graphs may be interpreted as schemes of cyclic molecules, in which two kinds of chemical bonds are present. In this case, the introduced Shannon entropies $\left(S_{\alpha },S_{\beta }\right)$ are seen as averaged uncertainties to find *α* or *β* cyclic sub-structures within the molecule. Another physical interpretation of the complete bi-colored graphs emerges when the vertices of the graph are treated as interacting physical entities. In this case, *α*-links may correspond to the attraction between the particles, and *β*-links may correspond, in turn, to repulsion between the interacting entities (electric or magnetic dipoles). Thus, the Shannon entropies $\left(S_{\alpha },S_{\beta }\right)$ are interpreted as averaged uncertainties to find *α* or *β* sub-structures, in which the aforementioned entities interact only by attraction or repulsion within an entire set of particles. We also plan to apply the developed approach to the analysis of the second-order phase transitions.

## Data Availability

The data are contained within the article.
